# Transmembrane Signal Transduction in Oocyte Maturation and Fertilization: Focusing on *Xenopus laevis* as a Model Animal

**DOI:** 10.3390/ijms16010114

**Published:** 2014-12-23

**Authors:** Ken-ichi Sato

**Affiliations:** Laboratory of Cell Signaling and Development, Department of Molecular Biosciences, Faculty of Life Sciences, Kyoto Sangyo University, Kamigamo-motoyama, Kita-ku, Kyoto 603-8555, Japan; E-Mail: kksato@cc.kyoto-su.ac.jp; Tel.: +81-75-705-2916

**Keywords:** fertilization, gamete interaction and fusion, membrane microdomains, oocyte maturation, signaling crosstalk, Src, uroplakin III

## Abstract

Fertilization is a cell biological phenomenon of crucial importance for the birth of new life in a variety of multicellular and sexual reproduction species such as algae, animal and plants. Fertilization involves a sequence of events, in which the female gamete “egg” and the male gamete “spermatozoon (sperm)” develop, acquire their functions, meet and fuse with each other, to initiate embryonic and zygotic development. Here, it will be briefly reviewed how oocyte cytoplasmic components are orchestrated to undergo hormone-induced oocyte maturation and sperm-induced activation of development. I then review how sperm-egg membrane interaction/fusion and activation of development in the fertilized egg are accomplished and regulated through egg coat- or egg plasma membrane-associated components, highlighting recent findings and future directions in the studies using *Xenopus laevis* as a model experimental animal.

## 1. Oocyte “Cytoplasmic” Signaling Events Associated with Meiotic Maturation and Fertilization, Focusing on Protein Phosphorylation

### 1.1. Meiosis and Oocyte Cytoplasmic Maturation

**Figure 1:**
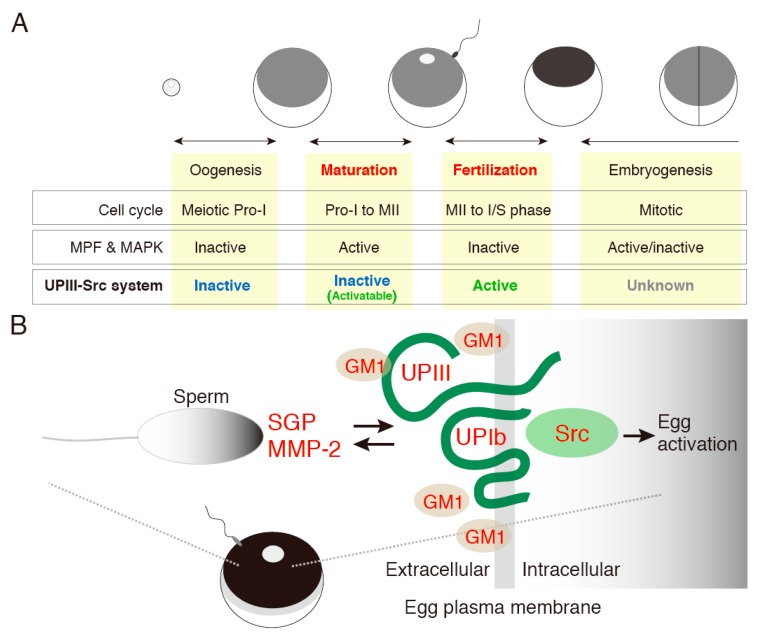
Egg membrane microdomain-associated UPIII-Src system in *Xenopus laevis*. (**A**) Shown is a schematic diagram for the cell cycle conditions, and the activation/inactivation switching of the MPF (maturation-promoting factor), MAPK (mitogen-activated protein kinase), and UPIII (uroplakin III)-Src system, in the course of oogenesis, oocyte maturation, fertilization, and early embryogenesis in *Xenopus laevis*; (**B**) Illustrated are sperm-egg interaction at the level of each gamete’s plasma membrane and its associated signaling events that involve SGP (sperm surface glygoprotein) and MMP-2 (matrix metalloproteinase-2) in sperm and UPIII/UPIb (uroplakin Ib) complex, GM1, and Src in eggs. For details, see main text.

Meiosis is the process by which diploid germ-line cell reduces their genetic material by half to generate haploid gametes. The haploid gametes, namely, egg and sperm, fuse with each other to create a genetically new, diploid individual. Oocyte maturation, which is undertaken during the meiotic cell cycle that is arrested at several stages depending on the species, has been studied extensively in many species of vertebrates and invertebrates [1,2,3,4,5,6]; studies on frog systems in particular have contributed to a detailed understanding of its biochemical nature [7,8]. In almost all vertebrates, the oocyte meiotic cell cycle starts during the fetal stage, but its first arrest occurs in the first meiotic prophase (Pro-I), which may last for several months or years in the follicular or ovarian microenvironment, depending on the species [9,10,11,12]. Hormone-stimulated resumption and further progression of the meiotic cell cycle are paused again, in many but not all species, at the stage of second meiotic metaphase II (MII). Different kinds of molecules, for example, extracellular ligands and oocyte membrane-surface receptors, and intracellular signaling molecules, are involved in these oocyte-specific dynamic cell cycle events (Figure 1A).

### 1.2. Maturation-Promoting Factor

MPF (maturation- or M-phase-promoting factor) is composed of a catalytic subunit, cyclin-dependent kinase 1 (Cdc2/CDK1), and a regulatory subunit, cyclin B; it acts as a key component in the maintenance of Pro-I arrest and its progression through MII arrest (for review: [13,14,15]) (Figure 1A). In activated MPF, CDK1 associates with cyclin B. The synthesis and degradation of cyclin B are finely regulated for the maintenance of MPF activity [16,17]. Cyclin B is accumulated in Pro-I-arrested oocytes due to the presence of early meiotic inhibitor 1 (Emi1), which inhibits anaphase-promoting complex/cyclosome (APC/C), a ubiquitin ligase complex responsible for the destruction of cyclin B [18]. The catalytic/kinase activity of MPF is also regulated by a complicated array of its interacting proteins, which include inhibitors of CDK (e.g., p21), CDK-stimulatory dual-specificity protein phosphatases (e.g., Cdc25), and CDK-inhibitory dual-specificity kinases (e.g., Wee1), some of which will be explained below.

### 1.3. Cyclic Nucleotides and Oocyte Cytoplasmic Maturation

In Pro-I-arrested oocytes, the concentrations of cAMP and cGMP are maintained at high levels by actions of the cumulus and granulosa cells in mammals and the follicle cells in amphibians; this is essential for the maintenance of meiotic arrest at the Pro-I stage [19,20,21,22]. The increased level of cGMP inactivates phosphodiesterase 3A (PDE3A) and prevents the hydrolysis of cAMP and thus a further increase in its level [22,23,24]. In Pro-I-arrested oocytes, high concentrations of cAMP activate protein kinase A (PKA), and activated PKA phosphorylates two CDK1 regulators, namely, Cdc25B phosphatase [25] and Wee1/Myt1 kinase [26,27]. The inactivation of Cdc25B and the activation of Wee1/Myt1 kinase ultimately inactivate MPF activity for the maintenance of meiotic arrest at the diplotene stage [26,28,29]. Luteinizing hormone (LH) released from surrounding granulosa cells act indirectly on oocytes to resume Pro-I arrest at the onset of puberty [9,30]. LH-mediated MAPK activation in granulosa cells interrupts the cell-oocyte communication and causes a decrease in cAMP and cGMP levels in oocytes [2,9,19]. A reduced level of oocyte cGMP causes the activation of PDE3A activity, which further reduces the oocyte cAMP level [31,32]. Net reduction of cAMP in oocytes inhibits PKA actions, while the dephospho-form of Cdc25B phosphatase becomes active [26]. On the other hand, the dephospho-form of Wee1/Myt1 kinase becomes inactive [2,26,29,33] and finally cancels the Pro-I arrest, which is morphologically characterized by germinal vesicle breakdown (GVBD) (for review [34]).

### 1.4. Mitogen-Activated Protein Kinase Cascade

Upon hormonal release from Pro-I arrest, maturing oocytes undergo activation of the MAPK cascade involving protein expression and/or enzymatic activation of Mos (MAPKKK), MEK (MAPKK), ERK (MAPK), and RSK. The activated MAPK contributes to proper organization of the metaphase spindle, allowing oocytes to accomplish progression of the first meiotic cell cycle [20] (Figure 1A). Oocytes remain arrested at Pro-I until the entire sister chromatids have properly attached to the bipolar spindle and aligned at the metaphase plate, where spindle assembly checkpoint (SAC) proteins e.g., Mad2, Bub1, and Bub3, function for all the required activities [12,35,36,37]. The SAC proteins function in accurate homologous chromosome segregation and in delaying the action of anaphase onset target APC/C [12,38,39]. The formation of a functional spindle and its migration correlate with progressively increased and sustained MPF activity [38,40]. Mos/MAPK activity is also important in microtubule reorganization and the positioning of the metaphase spindle to the oocyte cortex [41,42,43].

### 1.5. Cytostatic Factor and Secondary Cell Cycle Arrest in Meiotic Oocyte Maturation

At the end of the first meiosis, which is characterized by first polar body extrusion (the appearance of a white spot on the top of the animal hemisphere in the case of *Xenopus laevis* oocytes), MPF activity declines. After completion of the first meiosis, oocytes undergo some cytoplasmic changes and progress to secondary arrest at the metaphase of second meiosis (MII), with further high MPF activity until fertilization. Such stable MPF activity is maintained by CSF (cytostatic factor) activity [4,44,45] that involves the Mos-mediated MAPK pathway [46,47]. Emi1 and Emi2 (also called Erp1) are two members of the Emi/Erp family of proteins that act on CSF downstream of the MAPK pathway [48] and function in MII arrest [4,47,48,49]). A complex of dephosphorylated active Emi2 with Cdc20 inhibits APC/C, whose activation leads to the cancelation of MII arrest [47]. Recent studies have demonstrated that cell cycle arrest by Emi1 is distinct from the cytostatic factor-mediated cell cycle arrest in *Xenopus* MII oocytes [50] and that phosphorylation of Emi2/Erp1 is catalyzed by p90^Rsk^ [51,52]. In addition, transient activation of calcineurin, a protein serine/threonine phosphatase is required for the dephosphorylation-mediated inactivation of MPF [53,54]). MII arrest is regarded as a phenomenon in which all cytoplasmic and cell cycle events associated with meiotic oocyte maturation are accomplished and the oocytes become competent for fertilization, at least at the oocyte cytoplasm level (Figure 1A).

### 1.6. Sperm-Induced Resumption of Meiosis and Oocyte/Egg Activation

Sperm-egg interaction and fusion, that is, fertilization, promote the exit of the meiotically matured oocyte (unfertilized egg) from MII arrest. Sperm-mediated Ca^2+^ oscillations or Ca^2+^ release with wave-like propagation within the fertilized eggs activate calcium/calmodulin-dependent protein kinase II (CaMKII). Activated CaMKII and polo-like kinase simultaneously phosphorylate and inactivate Emi2 [4,47,55,56,57]). Cdc20, an APC/C activator that is sequestered by Emi2 at MII arrest, is released from the phosphorylated Emi2 and subsequently binds to APC/C, resulting in the forming of an active APC/C complex [58]. Activated APC/C induces the degradation of cyclin B, by which MPF activity declines, which might also inactivate the function of Mos [4,59,60]) (Figure 1A). Another mechanism of MII arrest involving Mos, MEK, ERK, and RSK, is modulated by a distinct mechanism. RSK or p90^Rsk^ induces SAC protein activation and thereby inhibits APC/C [4,61] to maintain MII arrest. At fertilization, Mos is degraded, by which its downstream kinases MEK/ERK/p90^Rsk^ are soon inactivated. At the end of this process, sister chromatids are segregated, the second polar body is extruded, and the first cleavage starts.

Sperm-induced release of MII arrest of an egg is also termed “egg activation”, which is characterized by many biochemical and cell biological reactions, for example, Ca^2+^ oscillations, cortical granules exocytosis, block to polyspermy, extrusion of polar body, formation of male and female pronuclei and their fusion, recruitment of maternal mRNAs, and initiation of DNA synthesis for mitotic divisions to unveil the complete developmental program [62,63,64]. The wave of Ca^2+^ initiates at the site of sperm binding/fusion and it is followed by a wave of intracellular Ca^2+^ traversing the entire cytoplasm of the egg [65,66,67,68,69,70]. The increase in the intracellular Ca^2+^ concentrations was reported in lysates of sea urchin eggs more than three quarters of a century ago [71], and thereafter, several excellent review articles have been published describing how an egg becomes developmentally activated at fertilization and how egg and sperm nuclei unite to form a zygote [63,66,72,73,74,75].

The postovulatory oocyte mimics the action of egg activation due to aging, increases cytoplasmic Ca^2+^, and induces exit from the MII arrest. However, it does not progress further and undergoes arrest again in a new metaphase-like stage called MIII in a few vertebrate species. The mechanism for MIII arrest remains poorly understood [76,77,78,79]. In aged eggs, insufficient Ca^2+^ release and sufficient CSF activity are still present to stabilize the residual or newly formed MPF activity, resulting in MIII arrest [78,80]. Some recent reports have demonstrated that the unfertilized aged eggs undergo apoptotic processes, such as cell swelling and caspase activation [81,82,83].

## 2. Plasma Membrane- and/or Egg Coat-Associated Events during Oocyte Maturation, Ovulation, and Fertilization

### 2.1. Mechanism of Progesterone and Its Receptor Interaction for Oocyte Maturation

Progesterone, the trigger of oocyte maturation in mammals and *Xenopus laevis*, is a canonical steroid hormone that provokes its cellular function through a genomic, that is, transcription-dependent, signaling mechanism. Accumulating evidence suggests, however, that the classical progesterone receptor mediates oocyte maturation in *Xenopus laevis* through a non-genomic, that is, transcription-independent and translation-dependent, signaling mechanism [84,85]. The classical progesterone receptor has long been thought to be a nuclear receptor that mediates the genomic cellular response. However, several lines of evidence demonstrate that the *Xenopus* oocyte progesterone receptor is excluded from the nucleus and acts as a cytoplasmic and/or membrane-associated receptor [86,87,88,89]). Another line of evidence has shown that androgen, another kind of steroid hormone, is produced in ovarian tissues in response to the enzymatic actions of oocyte CYP17, and it acts as the primary mediator of *Xenopus* oocyte maturation through its binding to the classical progesterone receptor [90,91].

### 2.2. Structure and Function of Vitelline Envelope/Membrane, and Other Egg Coats

The plasma membrane of fully grown immature oocytes is surrounded by glycoprotein-rich extracellular matrix called the vitelline envelope (VE)/membrane (VM) in invertebrates and amphibians, and by the zona pellucida (ZP) in mammals. Upon fertilization, this layer must be modified to prevent additional sperm binding and fusion in order to block polyspermy. The prevention of polyspermy is accomplished in part through Ca^2+^-dependent cortical granule exocytosis [92,93]. Upon egg activation, cortical granules fuse with the oocyte plasma membrane and release their contents into the perivitelline space, which results in the formation of fertilization envelope (FE).

The vitelline membrane of *Xenopus laevis* oocyte has a thickness of 5–10 nm and is composed of some glycoproteins, which include gp37 (also termed as ZPB), gp41 (ZPC), gp69/64 (ZPA), gp80 (ZPD), and gp120 (ZPY), all of which are synthesized within growing oocytes and are *N*-glycosylated with mannose moieties, which have been reported to be important for fertilization [94]. It should be noted that gp69/64, gp37, and gp41 are amphibian homologs of the mammalian zona pellucida proteins ZP2, ZPB pseudogene, and ZP3, respectively [95]. Goudet *et al.* [95] also demonstrated that *Xenopus* species do not possess ZP1 homologue. Maturing and ovulating oocytes are exposed to the activity of the tryptic protease oviductin, which is secreted from epithelial cells in the pars recta of oviduct, by which gp43, a precursor of gp41, is converted to gp41 [96]. This proteolytic conversion allows sperm to bind to the vitelline membrane at fertilization. In addition, two pars recta components are known to be secreted, and attach the vitelline membrane during passage of oocytes in the oviduct: the 105-kDa protein that contributes to FE formation [97] and the acrosome reaction-inducing substance in *Xenopus* (ARISX) [98]. In the subsequent area of the oviduct, the pars convoluta, oocytes are further coated with jelly layers of J1, J2, and J3, which are composed of carbohydrates and proteins [99,100]. Finally, jellied oocytes are supplied with allurin, which contributes to sperm’s chemotactic movement toward the spawned egg [101,102]. These successive modifications involving the vitelline membrane and jelly layers collectively constitute a mechanism that leads to the acquisition of fertilization competency at the level of “maturation of egg/oocyte coat”.

### 2.3. Sperm Plasma Membrane-Associated Components

As mentioned above, cellular components that are involved in the acquisition of fertilization competency in maturing and ovulating oocytes and their roles in fertilization and activation of development have been well documented at the level of cytoplasm and egg/oocyte coat in *Xenopus laevis* (*i.e.*, jelly layers and vitelline membrane). After passing through the jelly layers and vitelline membrane, fertilizing sperm must adhere to and penetrate into the plasma membrane of egg to complete fertilization. However, until recently, little has been known about what kind of molecular machinery is involved in these fundamental but complicated processes.

On the sperm side, a study using a monoclonal antibody has demonstrated that glycoproteins of 60–150 and 20–28 kDa on SDS-PAGE, collectively called sperm surface glycoprotein (SGP), are candidates as the binding partner to the egg plasma membrane at fertilization [103] (Figure 1B). A monoclonal antibody (named 2A3D9) that has been raised against the sperm-derived membrane proteins has been shown to inhibit normal sperm-egg interaction [103]. Immunochemical detection of SGP with 2A3D9 on the egg surface treated with sperm-derived membrane fraction has demonstrated that SGP binding to the egg surface is limited to the animal hemisphere. This observation suggests that SGP and its binding to an unknown egg partner account for the well-known phenomenon that sperm entry occurs only in the animal hemisphere in *Xenopus laevis*. Study on SGP has also demonstrated that it binds to the vitelline membrane gp37, the *Xenopus* homolog of mammalian ZP1 [104]. Thus, it is possible that SGP is involved in two distinct processes for sperm interaction with egg: vitelline membrane- and plasma membrane-interactions. It should also be noted that interaction between sperm and egg vitelline membrane has been shown to involve gp69/gp64 [105,106] and gp41 [107].

A more recent study has shown that SGP physically interacts with another sperm protein, matrix metalloproteinase 2 (MMP-2), on the sperm membrane [108] (Figure 1B). This physical interaction was demonstrated in a co-immunoprecipitation study. It was suggested that MMP-2, which by itself has neither a transmembrane nor a membrane-anchoring structure, interacts with SGP, by which it is allowed to localize to the sperm membrane and gains the potential to interact with the egg plasma membrane. MMP-2 has a canonical MMP domain and pharmacological experiments have shown that the enzymatic activity seems to be important for sperm to bind and/or pass through the egg vitelline membrane. MMP-2 has another functional domain named the hemopexin (HPX) domain, which contains a disintegrin sequence Arg-Gly-Glu that is known to interact with and activate integrin family proteins. A synthetic peptide that corresponds to a part of the HPX domain, which contains the Arg-Gly-Glu sequence, when applied to unfertilized *Xenopus* eggs, is shown to cause egg activation accompanied by intracellular Ca^2+^ release within the egg [108]. Interestingly, the peptide-induced egg activation requires the egg plasma membrane potential of less than zero (0 V) [108].

This phenomenon resembles the sperm-induced egg activation at fertilization, where sperm can activate an egg only when the membrane potential of the egg is less than zero [109,110]. Studies by other groups have demonstrated that the synthetic Arg-Gly-Asp peptide can induce egg activation [111,112], but has no sensitivity to membrane potential, and that a synthetic peptide covering the disintegrin motif (Lys-Thr-Cys) of sperm xMDC16 protein is capable of activating egg [113]. However, xMDC16 is expressed mainly in the midpiece, not the acrosomal region, of sperm [114]. Thus, the sperm MMP-2 interaction with the egg plasma membrane offers the first candidate mechanism for explaining the long-suspected voltage-dependent nature of the sperm interaction with the egg plasma membrane and subsequent egg activation in *Xenopus laevis*.

### 2.4. Egg/Oocyte Plasma Membrane-Associated Components

On the egg side, studies on the plasma membrane-associated tyrosine kinase Src have shed light on the identity of the molecular machinery that is responsible for gamete interaction and possibly fusion (Figure 1B). It was 1996 when we reported that the egg-associated tyrosine kinase Src (called p57 kinase or *Xenopus* tyrosine kinase) is activated within minutes of *in vitro* fertilization of *Xenopus laevis* [115]. The activated Src has been shown to contribute to phosphorylation and activation of phospholipase Cγ, by which it also contributes to inositol 1,4,5-trisphosphate-dependent intracellular Ca^2+^ release within the fertilized eggs [116,117,118,119,120]. Other downstream targets, that is, phosphorylation substrates, of the activated Src include the RNA-binding protein heterogeneous nuclear ribonucleoprotein K [121]), the Src homology 2-containing adaptor protein Shc [122], and phosphatidylinositol 3-kinase [123], although the physiological outcomes for these interactions remain unclear [124].

Src family tyrosine kinases (SFKs) play important roles in sperm-induced Ca^2+^ response in several species, for example, in starfish [125,126,127,128], Fyn kinase in sea urchin eggs [129] and in rat eggs [130], unknown Src-related kinase in ascidian eggs [131], and Src in frog eggs [115,120]. In mouse eggs, although the expression of Src-related tyrosine kinase (e.g., Lck, Src) has been reported [132,133], its activity is not sufficient or required for fertilization-induced Ca^2+^ oscillation [132]. In mammals, PLC activity is sufficiently high in sperm, which is why even a single sperm equivalent of PLC can generate sufficient IP_3_ when introduced into the egg cytoplasm [134]. The ζ-isoform of PLC in sperm has been characterized as a soluble sperm factor that evokes Ca^2+^ oscillations in eggs of several mammals, for example, mouse, bovine, and human [135,136,137,138]. Other reports have demonstrated that other proteins, such as truncated c-Kit tyrosine kinase [139] or postacrosomal WW binding protein [140] in mammals, citrate synthase in newt [141,142], and citrate synthase and aconitate hydratase [143] in bird, could also be an essential factors for Ca^2+^ oscillations or a single Ca^2+^ spike in fertilized eggs of these species.

Upstream interaction partner, for example, sperm-dependent kinase regulator, for Src has also been identified from comparative phosphorylation studies of unfertilized and fertilized *Xenopus* eggs. Uroplakin III (UPIII), a 30-kDa and glycosylated single-transmembrane protein, was initially identified as a predominantly tyrosine-phosphorylated protein that localizes to low-density, detergent-insoluble membrane (LD-DIM) fractions of fertilized *Xenopus* eggs [144]) (Figure 1B). LD-DIM represents a membrane subdomain or microdomain (MD) that is enriched in membrane lipid components such as cholesterol and sphingolipids (-containing substances), and a specific subset of proteins. Pharmacological and biochemical experiments have demonstrated that MD of unfertilized *Xenopus* egg acts as a platform for sperm-induced Src tyrosine kinase signaling at fertilization [145,146,147]. Src is also enriched in the egg MD fraction [146].

Further study of UPIII has shown that it has sperm-interacting function as a target of sperm protease [148]. *Xenopus* fertilization requires tryptic protease activity of sperm at the level of gamete plasma membrane interaction [109,149]. Its pharmacological inhibition results in a failure of sperm-induced activation of Src and embryonic development [148]). The sperm protease has been purified to near homogeneity by biochemical and chromatographic fractionation [149]; however, its molecular identity and subcellular localization (suspected to be the acrosomal head of sperm) remains unclear.

UPIII has a binding partner, uroplakin Ib, a tetraspanin transmembrane UP family protein that contributes to UPIII’s exit from the endoplasmic reticulum and membrane localization [150,151]) (Figure 1B). The UPIII-UPIb complex on the egg surface physically associates with the ganglioside GM1, a well-known component of MD [145,150] (Figure 1B). MMP-2, a sperm component involved in egg membrane interaction, was shown to directly bind to GM1 *in vitro* [108]. The ganglioside GM1 has a negative charge in its molecular structure; on the other hand, the HPX domain of sperm MMP-2 has a positive charge in its structure, suggesting that such an electrically complementary nature of these molecules could serve as a basis for their interaction, and that this interaction could provide the means for a voltage-dependent gamete interaction at the plasma membrane level (Figure 1B).

The mechanism by which sperm-induced proteolysis of UPIII induces activation of the egg cytoplasmic Src remains unclear. Studies in mammalian cells have demonstrated that proteolysis of the extracellular domain of cell surface seven-transmembrane receptor (e.g., receptor for thrombin, protease receptor) [152,153] or single transmembrane protein (e.g., CD44, Notch) [154,155] is a mechanism for triggering intracellular signal transduction. One possible intermediate for Src activation in response to UPIII proteolysis is phosphatidylinositol 3,4,5-triphosphate, an enzymatic product of PI 3-kinase [123]. More recently, possible involvement of phosphatidic acid (PA), an enzymatic product of phospholipase D, in sperm-induced activation of Src has been documented [156]. It is interesting to note that the plasma membrane of not only fertilized egg but also of fertilizing sperm has been postulated as a resource for PA; the latter possibility suggests an egg activation mechanism by direct introduction of a sperm-derived factor [120,156].

### 2.5. Physiological Function of Egg MD (Microdomain) and Its Acquisition during Oocyte Maturation

More recent study has demonstrated that the UPIII-Src system in egg MD acts not only for receiving and transmitting egg activation signals from fertilizing sperm, but also for “activating” the fertilizing sperm through their membrane interaction [157]. Experiments using isolated MD in unfertilized eggs have shown that the MD fraction is capable of interacting with fertilizing sperm, by which sperm become able to fertilize eggs that are pretreated with an antibody that binds to the extracellular domain of UPIII and inhibits sperm-induced proteolysis of UPIII and subsequent Src-dependent egg activation events. The results suggest that the exogenously added MD fraction “activates” sperm; otherwise, fertilization of the antibody-treated eggs is not possible. Such activation mechanism of the MD-treated sperm involves proteolysis of UPIII in the MD fraction and sperm protein kinase activity, the latter of which has been suggested by pharmacological inhibition studies [157]. More detailed study to explore the membrane interaction between MD and sperm, for example, possible ligand function of the partially proteolysed UPIII extracellular domain and the molecular identity of sperm protein kinase that is involved in the MD-induced “activation” of sperm, is awaited.

Another line of recent studies has demonstrated that the UPIII-Src system acquires its functional competency, that is, the ability to receive the sperm signal and to transmit it to the egg cytoplasm, during hormone-induced oocyte maturation [157] (Figure 1A). Immunochemical and surface biotinylation studies have shown that UPIII is expressed in ovarian immature oocytes from the beginning of oogenesis. Indirect immunofluorescent studies have shown, however, that the UPIII extracellular domain becomes more accessible to the anti-UPIII extracellular domain antibody (as mentioned above) after hormonal treatment of oocytes for maturation. *In vitro* reconstitution experiments using isolated MD fractions of sperm-induced UPIII proteolysis and Src activation have also demonstrated that the responsiveness of MD of fully grown immature oocytes to sperm is much weaker than that of *in vitro* maturing oocytes or of ovulated unfertilized eggs [157]. The aforementioned sperm “activating” property is also weak in MD of immature oocytes. Taken these findings together, the MD-associated UPIII-Src system in MD seems to represent the first example of molecular machinery that undergoes maturation at the oocyte/egg plasma membrane level in *Xenopus laevis*.

## 3. Future Perspective

Molecular mechanism of gamete interaction/fusion and subsequent signaling events associated with activation of embryonic development has long been one of the most important themes in the study of the physiology of fertilization [158,159]. Study using *Xenopus laevis* oocyte and egg is advantageous for biochemical and cell biological experiments to elucidate protein functions. By employing this excellent animal model, a number of pioneering findings have been made in the field of oocyte maturation at the oocyte cytoplasm and egg/oocyte coat levels. On the other hand, *Xenopus laevis* oocyte and egg have not been effectively employed as an experimental platform to characterize plasma membrane-associated components that act in gamete interaction and signaling. Against this background, a mouse oocyte and egg system, which has an advantage for conducting gene knockout studies, has recently provided both sperm and egg components that act in gamete interaction in the plasma membrane. In the mouse, egg membrane components called exosomes that contain CD9, the tetraspanin protein essential for mouse gamete fusion on the egg side [160,161,162], have been shown to interact with or transfer to fertilizing sperm before sperm contact the egg plasma membrane [163,164], so that the sperm’s ability to fuse with the egg becomes fully competent [165]. This observation and accompanying hypothesis, however, have been challenged by some other studies [166,167]. In addition, it is unknown when and how such an exosome-oriented egg system for gamete fusion is established [168]. On the sperm side, IZUMO1 has been shown to be essential for gamete interaction and fusion [169]. It has been shown that IZOMO1 does not bind to CD9 [170], but it does to Juno, the folate receptor 4 in the egg [171]. Knockout mice producing Juno-deficient eggs are infertile and this infertility has been shown to be due to the failure of the eggs to bind to and fuse with fertilizing sperm [171]. In *Arabidopsis*, mutual signaling between egg cell-derived EGG CELL 1 protein [172] and sperm has been shown to be responsible for the cell surface presentation of GCS1/HAP2 that is essential for the gamete fusion [173]. These reports suggest that gamete plasma membrane interaction acts as a trigger of mutual signal transduction in a wide variety of organisms. As mentioned above, much progress has been achieved in terms of our understanding of gamete plasma membrane-associated mechanism of gamete interaction and signaling for fertilization in the frog, *Xenopus laevis* (see Section 2). Further study should be designed to determine whether knockout frog that produces a certain gene-deficient gamete (e.g., MMP-2 in sperm, UPIII in egg) is fertile or not. It would also be interesting to examine whether the mammalian components that have been shown to be essential for gamete interaction and signaling (*i.e.*, IZUMO1 in sperm, CD9 and Juno in egg) are also essential for fertilization in this species. Such combinational studies of fertilization using biochemical and cell biological approaches and genetic methodologies, which have become available very recently (e.g., TALEN-mediated genome editing technology) [174,175,176], should provide us with a deeper understanding of the molecular mechanism of oocyte maturation and fertilization in the frog, *Xenopus laevis*, and its related species, *Xenopus tropicalis*.
